# Predictive significance of glycolysis-associated lncRNA profiles in colorectal cancer progression

**DOI:** 10.1186/s12920-024-01862-2

**Published:** 2024-04-29

**Authors:** Rui Mao, Chenxin Xu, Quanzheng Zhang, Zheng Wang, Yanjun Liu, Yurui Peng, Ming Li

**Affiliations:** 1 Hunan Key Laboratory of Aging Biology, Xiangya Hospital, Central South University, Changsha, China; 2 Center of Gastrointestinal and Minimally Invasive Surgery, Department of General Surgery, The Second Affiliated Hospital of Chengdu, The Third People’s Hospital of Chengdu, Affiliated Hospital of Southwest Jiaotong University, Chongqing Medical University, NO.82 Qinglong Road, Chengdu, Sichuan, 610031 China; 3 Center of Obesity and Metabolism disease, Department of General surgery, The Second Affiliated Hospital of Chengdu, The Third People’s Hospital of Chengdu, Affiliated Hospital of Southwest Jiaotong University, Chongqing Medical University, Chengdu, 610031 China; 4 Department of Critical Care Medicine, Chengdu Third People’s Hospital, Chengdu, 610031 China; 5 Department of Colorectal Surgery, National Clinical Research Center for Cancer, Cancer Hospital, National Cancer Center, Chinese Academy of Medical Sciences and Peking Union Medical College, Beijing, China; 6 Department of hepatobiliary surgery, The Second Affiliated Hospital of Chengdu, The Third People’s Hospital of Chengdu, Affiliated Hospital of Southwest Jiaotong University, Chongqing Medical University, NO.82 Qinglong Road, Chengdu, Sichuan, 610031 China

**Keywords:** Warburg effect, Colorectal cancer, lncRNA, Prognostic signature, Nomogram

## Abstract

**Background:**

The Warburg effect is a hallmark characteristic of colorectal cancer (CRC). Despite extensive research, the role of long non-coding RNAs (lncRNAs) in influencing the Warburg effect remains incompletely understood. Our study aims to identify lncRNAs that may modulate the Warburg effect by functioning as competing endogenous RNAs (ceRNAs).

**Methods:**

Utilizing bioinformatics approaches, we extracted glycolysis-associated gene data from the Kyoto Encyclopedia of Genes and Genomes (KEGG) and identified 101 glycolysis-related lncRNAs in CRC. We employed Univariable Cox regression, Least Absolute Shrinkage and Selection Operator (LASSO) regression analysis, and Multivariable Cox regression to develop a prognostic model comprising four glycolysis-linked lncRNAs. We then constructed a prognostic nomogram integrating this lncRNA model with other relevant clinical parameters.

**Results:**

The prognostic efficacy of our four-lncRNA signature and its associated nomogram was validated in both training and validation cohorts. Functional assays demonstrated significant glycolysis and hexokinase II (HK2) inhibition following the silencing of RUNDC3A − AS1, a key lncRNA in our prognostic signature, highlighting its regulatory importance in the Warburg effect.

**Conclusions:**

Our research illuminates the critical role of glycolysis-centric lncRNAs in CRC. The developed prognostic model and nomogram underscore the pivotal prognostic and regulatory significance of the lncRNA RUNDC3A − AS1 in the Warburg effect in colorectal cancer.

**Supplementary Information:**

The online version contains supplementary material available at 10.1186/s12920-024-01862-2.

## Introduction

Colorectal cancer (CRC) occupies a significant position among gastrointestinal malignancies, ranking as the fourth most common cause of cancer-related deaths worldwide. Annually, it accounts for over 550,000 fatalities and registers more than 1 million new cases [1]. Despite considerable research endeavors, the complex etiology of CRC continues to be only partially understood, thereby limiting the potential for early diagnosis and effective intervention [2]. As a result, the prognosis for many CRC patients remains relatively poor [3]. This underscores the urgent need for in-depth research into CRC’s multifaceted mechanisms, aimed at discovering innovative diagnostic and prognostic biomarkers.

A hallmark of cancerous cells is their relentless proliferation, not merely due to unchecked growth but also because of metabolic alterations. These adaptations are crucial for meeting the energy and macromolecular demands of cells, particularly in the hypoxic and nutrient-deprived microenvironment of tumors. Such metabolic flexibility, known as metabolic reprogramming, has recently been recognized as a critical aspect of cancer biology. In this context, aerobic glycolysis, famously known as the Warburg effect, plays a pivotal role in this metabolic transformation [4]. The essence of the Warburg effect is characterized by the abnormal activity of key glycolytic enzymes [5], notably hexokinase II (HK2). HK2, functioning downstream of oncogenes, significantly contributes to the maintenance of this altered metabolic state [6].Long non-coding RNAs (lncRNAs), which are transcripts exceeding 200 nucleotides, uniquely intervene in cellular processes without encoding proteins [7]. Their pivotal role in the evolution and progression of colorectal cancer (CRC) has been established, particularly as competing endogenous RNAs (ceRNAs). These ceRNAs modulate target gene expression by sponging microRNAs (miRNAs) [8]. Research by Meng et al. has shown that the lncRNA LINC00525 influences the Warburg effect in CRC by activating Hypoxia-Inducible Factor 1-alpha (HIF-1α) through the miR-338-3p/UBE2Q1/β-catenin signaling pathway [9]. While Zhu J and colleagues have linked glycolysis-related genes with CRC prognosis [10], a comprehensive catalog of lncRNAs driving the Warburg effect is still lacking. Furthermore, prognostic assessments using glycolysis-related lncRNAs in CRC have not been adequately explored.

In our study, we extracted a list of glycolysis-related genes from the KEGG database and integrated gene expression profiles with relevant clinical data of CRC patients from The Cancer Genome Atlas (TCGA). Utilizing Gene Set Variation Analysis (GSVA), we determined the enrichment scores of the glycolysis pathway, thereby revealing the relationship between glycolytic activity and CRC prognosis. Building on this foundation, we developed a ceRNA network to identify glycolysis-related lncRNAs, leading to the creation and validation of a glycolysis-focused prognostic signature. We highlighted a specific lncRNA from this signature to emphasize its significant role in CRC glycolysis, suggesting new potential targets for therapeutic intervention in CRC.

## Materials and methods

### Data acquisition and preprocessing

RNA expression datasets, accompanied by clinical data for CRC patients, were sourced from The Cancer Genome Atlas (TCGA) database (https://portal.gdc.cancer.gov/). Sample inclusion criteria were as follows: (1) histologically confirmed CRC; (2) mRNA expression profile data and clinical information were available simultaneously; and (3) only samples with an overall survival (OS) time longer than 30 days were involved when survival analysis was performed. LncRNAs and mRNAs quantification followed the reference catalog provided by the GENCODE (GRCh38) from the Genome Research Project of ENCyclopedia of DNA Elements (https://www.gencodegenes.org/). Long non-coding RNA gene annotation The reference table is provided in the supplementary material. Glycolysis-centric genes were collated from the KEGG’s hsa00010 gene dataset (https://www.genome.jp/kegg/pathway/hsa/hsa00010.html). The “caret” package in R was employed to randomly bifurcate eligible patients into two cohorts: Training and Validation.

### Differentially expressed analysis

Employing the R “limma” package, we discerned differentially expressed lncRNAs (DElncRNAs), miRNAs (DEmiRNAs), and glycolysis-associated mRNAs (DEmRNAs) between CRC and adjacent non-tumor tissues. The delineation criteria were an absolute logFC value of ≥ 1 and an adjusted P-value of < 0.05. Resultant differential expression patterns were visualized via heatmaps, generated using the “pheatmap” R package.

### Gene set variation analysis (GSVA)

GSVA represents a non-parametric and unsupervised approach to gene set enrichment, facilitating the estimation of gene set activity specific to individual samples based on gene expression data. Contrasting with conventional enrichment analyses like Gene Set Enrichment Analysis (GSEA), GSVA uniquely operates on an individual sample basis, thus offering a novel methodology to assess variations in gene set expression within a singular sample context. GSVA was conducted utilizing the “GSVA” R package. Post-analysis, patient data was bifurcated, based on the median enrichment score of the KEGG glycolysis pathway, into two distinct clusters.

### The construction of the ceRNA network

We consulted the miRTarBase (http://mirtarbase.mbc.nctu.edu.tw/), miRDB (http://www.mirdb.org/), and TargetScan (http://www.targetscan.org/) databases to pinpoint miRNAs potentially modulating glycolysis-related DEmRNAs. Only miRNAs concurrently acknowledged across these three databases were deemed as “predicted miRNAs.” The StarBase platform (http://starbase.sysu.edu.cn/) facilitated the identification of interactions between lncRNAs and our predicted miRNAs, yielding a subset of anticipated lncRNAs. Ensuing this, we juxtaposed these predicted entities with DEmiRNAs and DElncRNAs from TCGA CRC datasets, retaining only coinciding miRNAs, lncRNAs, and their interaction duos for subsequent analyses. The curated glycolysis-focused ceRNA interaction network was visualized via the Cytoscape 3.6.1 software.

### The construction of the glycolysis-related lncRNA-based prognostic signature

Univariate and multivariate Cox regression, LASSO analyses were used to select the independent risk glycolysis-related lncRNAs. Multivariate COX regression was used to identify corresponding coefficients of CRC prognostic signature which was calculated according to the following formula:

$$\eqalign{{\rm{Risk}}{\mkern 1mu} {\rm{score}}{\mkern 1mu} {\rm{ = }}{\mkern 1mu} & \sum r egression\,coefficient\left( {lncRN{A_i}} \right) \cr & \times \,expression\,value\left( {lncRN{A_i}} \right) \cr} $$,

using “glment”, “survminer” and “survival” packages of R. According to the equation above, risk score of each patient was calculated separately in the training and validation cohorts. Patients were subsequently divided into high- and low-risk groups with the median risk score as the cut-off point. Kaplan-Meier curves and the log-rank test were used to compare the survival outcomes of the two groups. Receiver operating characteristic (ROC) curve analysis and ﻿Harrell’s concordance index were employed to assess the accuracy and precision of the survival predictions according to the risk scores.

### Development and validation of the prognostic nomogram with signature and clinicopathological parameters

Risk score and the clinicopathological parameters are selected as candidate predictors in this model. Univariate and Multivariate COX regressions were analysed. All the identified independent prognostic parameters were utilized to develop a prognostic nomogram for predicting the 1-, 3-, and 5-year survival outcomes of CRC patients using the “rms” package of R. Calibration plots at 1-, 3-, and 5-year were constructed to graphically evaluate the discriminative ability of the nomogram. Decision curve analysis (DCA) was performed using the code downloaded from MSKCC (https://www.mskcc.org/). The discrimination performance of the nomogram was quantitatively assessed by the concordance index, ROC curve, and DCA.

### RNA extraction and quantitative reverse transcription PCR (qRT-PCR) analysis

TRIzol reagent (Invitrogen, Carlsbad, CA, USA) was used to isolate total RNA. Then the total RNA was reverse transcribed into cDNA with random primers using the Transcriptor First Strand cDNA Synthesis Kit (Roche, Penzberg, Germany). The relative quantification of lncRNA RUNDC3A − AS and mRNA HK2 was examined using the SYBR green PCR Master Mix (Qiagen, Hilden, Germany), with internal GAPDH as control. The data were processed using a 2 − ΔΔCt method. All reactions were run in triplicate independently. The primers used are presented in supplementary Table S1.

### Cell culture and reagents

CRC cell lines, including SW480, SW620 were obtained from Cell Bank of Type Culture Collection, Chinese Academy of Sciences (Shanghai, China). SW480 and SW620 cells were maintained in RPMI 1640 medium (Invitrogen) supplemented with 10% FBS at 37 °C in a humidified atmosphere of 5% CO2. Knockdown experiments were conducted using siRNAs targeting lncRNA RUNDC3A − AS1 and negative control (siNC) with Lipofectamine RNAiMAX reagent (Invitrogen), according to manufacturer’s instructions. The sequence of siRNA is 5’-GTCTCTACATTGATTCAGATTTG-3’.

### Glucose consumption and lactate production assays

Glucose was quantified by glucose assay kits (Applygen Technologies, Beijing, China) and glucose consumption was calculated by subtracting the amount of glucose present in the supernatants of cell culture medium. Lactic acid produced in the culture medium was measured using an assay kit (Nanjing Jiancheng Bioengineering Institute, Nanjing, China) according to the manufacturer’s protocol.

### Protein extraction and western-blot analysis

SW480 and SW460 cells in 6-well plates were lysed with radio immunoprecipitation assay (RIPA) buffer (Thermo Scientific, Waltham, MA, USA) with a protease inhibitor cocktail (Roche, IN, USA). Equal amounts of protein were separated by 10% SDS-PAGE gel electrophoresis and transferred onto a polyvinylidene difluoride membrane. The membranes were probed with antibodies anti-HK2 (ab104836; Abcam) or GAPDH (ab9484; Abcam). Then, the probed membranes were incubated with horseradish peroxidase‐conjugated secondary anti-body. To visualize signals, enhanced chemiluminescence (ECL) substrates (Bio-Rad) was conducted, with GAPDH as an endogenous protein for normalization.

### Colony formation assays

Cell colony formation rate was detected using colony formation assays. 0.5 × 103 colorectal cancer cells were plated in a 60-mm plate and cultured for 7 days. Then, 10% formaldehyde was used for fixing plates for 5 min and 1% crystal violet was used for stained for 30 s. Finally, counting the number of colonies.

### Statistical analysis

Statistical analyses were performed with R Project. We checked the distributions of metrological data fitted the normal distribution using the Kolmogorov–Smirnov test. Comparisons between the two groups were performed using the non-parametric Mann-Whitney U-test and t-test, where applicable. Chi-square test was used to detect the differences in the clinicopathologic characteristics of patients in two different groups. Survival was analyzed using Kaplan-Meier survival analysis. A two-side test *P* < 0.05 was indicated statistical significance.

## Results

### Dysregulated glycolysis plays an important role in the development of CRC

**Fig. 1:**
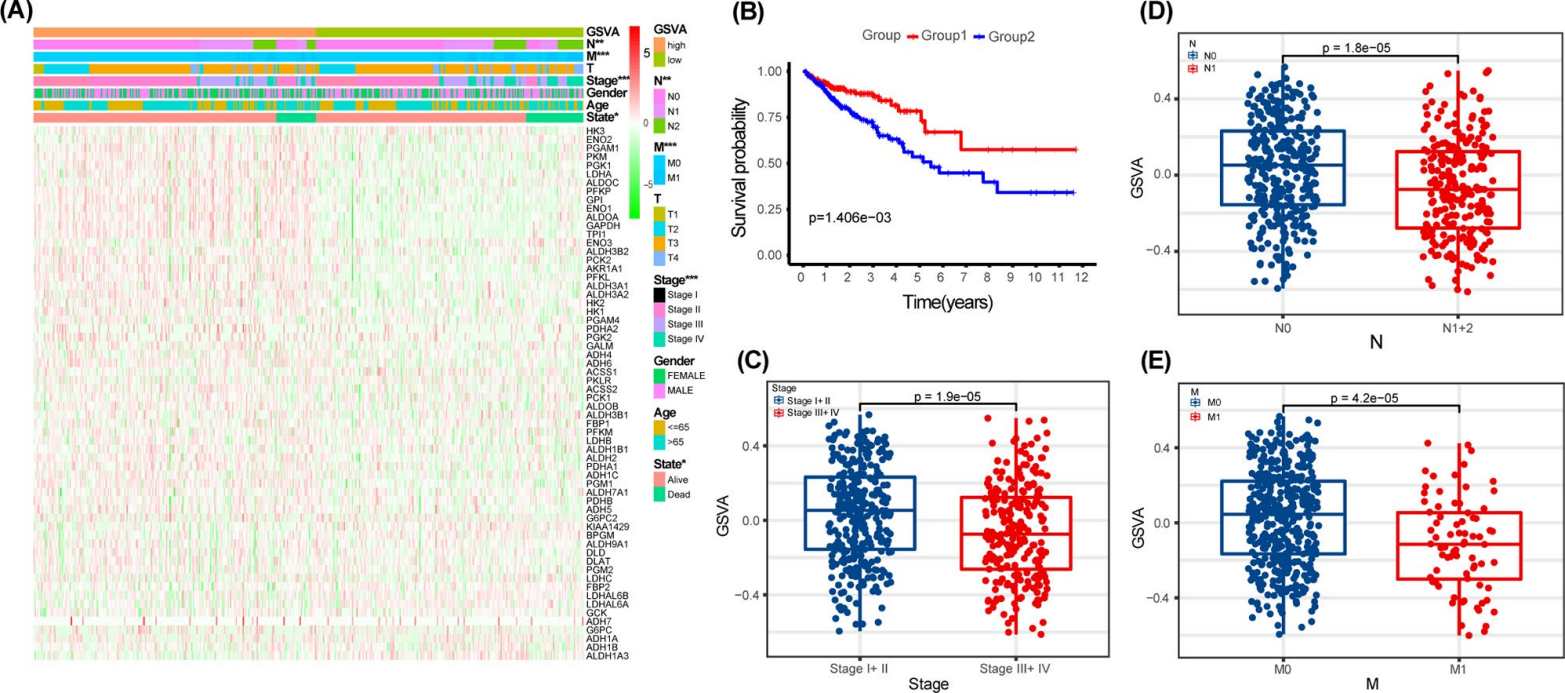
Glycolysis pathway plays an important role in CRC. **(A)** The heatmap of glycolysis-related genes expression in TCGA datasets ranked based on GSVA score of glycolysis pathway and the relationship between the GSVA score of glycolysis pathway and CRC-related clinical characteristics. *p < 0.05; **p < 0.01; **p < 0.001. **(B)** Kaplan–Meier curves of OS in TCGA-CRC patients who were were divided into two groups based on the glycolysis pathway GSVA score. **(C-E)** Patients with different clinical characteristics showed significantly different glycolysis pathway GSVA scores

First, we calculated the glycolysis pathway enrichment score of every CRC patient in the TCGA RNA-seq dataset by using GSVA method to explore the association of glycolysis activity and clinical outcome. Patients were divided into two groups with the median score of glycolysis pathway as the cutoff point. Patients in group1 had a higher enrichment score of glycolysis pathway than patients in group2. The heatmap of the two groups showed significant differences in glycolysis-related genes expression (Fig. 1A). Subsequently, we performed Chi-square tests to see if there exist any differences in clinical characteristics between these 2 groups of patients. The results showed that patients in group1 had the characteristics of lower stage grade, lower N, M stage, and higher possibility of being alive. Moreover, Kaplan-Meier curve analysis revealed that the OS time of patients in group1 was significantly longer than that of patients in group2 (Fig. 1B). Wilcox test was performed to further detect the difference in GSVA enrichment score between patients with different clinical characteristics (Fig. 1C-E). The results suggested lower glycolysis pathway enrichment score was associated with a higher tumor stage, N and M stage (*p* < 0.05). These results indicated that glycolysis GSVA enrichment score was closely correlated with clinical outcomes of patients with CRC, suggesting an essential biological role of dysregulated glycolysis in CRC development.

### Construction of the CeRNA network

A total of 568 CRC samples and 44 adjacent normal samples were downloaded to screen DElncRNAs and DEmRNAs; 446 CRC samples and 45 adjacent normal samples were utilized to screen DEmiRNAs. 62 glycolysis-related genes were extracted from KEGG glycolysis gene set, and 11 of which were differentially expressed in CRC samples. 2550 DElncRNAs and 212 DEmiRNAs were screened from TCGA CRC datasets.

**Fig. 2:**
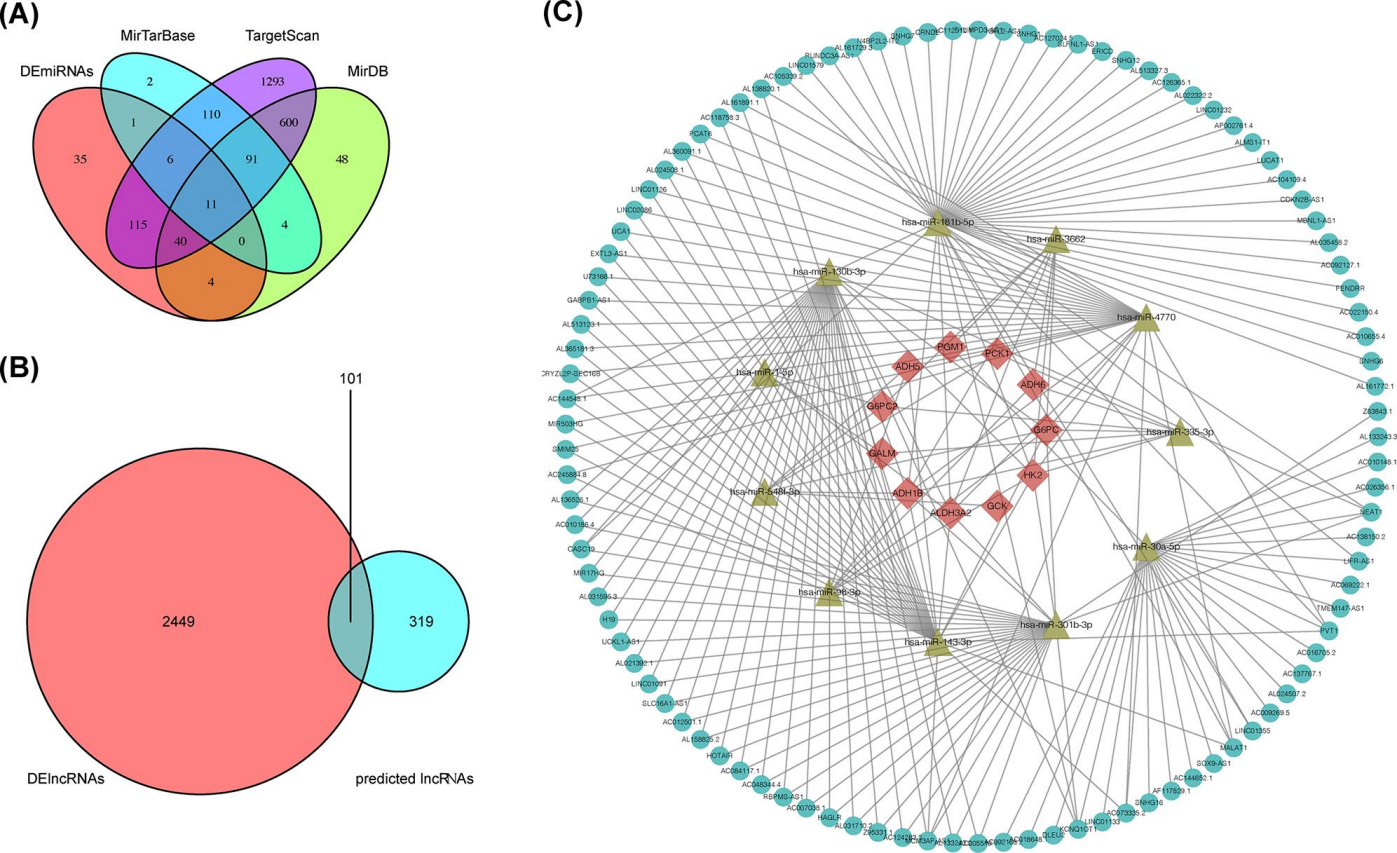
Screen out glycolysis-related lncRNAs by constructed ceRNA network. **(A)** 11 target miRNAs were obtained by taking the intersection of the predicted-miRNAs and DEmiRNAs; **(B)** 101 glycolysis-related lncRNAs were obtained by taking the intersection of the predicted-lncRNAs and DElncRNAs; **(C)** Construction of a ceRNA network

We then tried to find predicted targeting miRNAs of the glycolysis-related DEmRNAs from TargetScan, miRDB, and miRTarBase databases. After intersecting with the DEmiRNAs, only 11 miRNAs remained (Fig. 2A). Subsequently, 319 target lncRNAs were predicted for these 11 miRNAs by StarBase databases, which were then overlapped with 2449 DElncRNAs collected from the TCGA, resulting in 101 lncRNAs shared (Fig. 2B). The top 10 up-regulated and down-regualted Warburg-effect related lncRNAs are showed in Tables S1 and Table S2 respectively. Based on the combination of the above data, we established the ceRNA network using cytscope (Fig. 2C).

### Establishment of glycolysis-related lncRNA signature

**Fig. 3:**
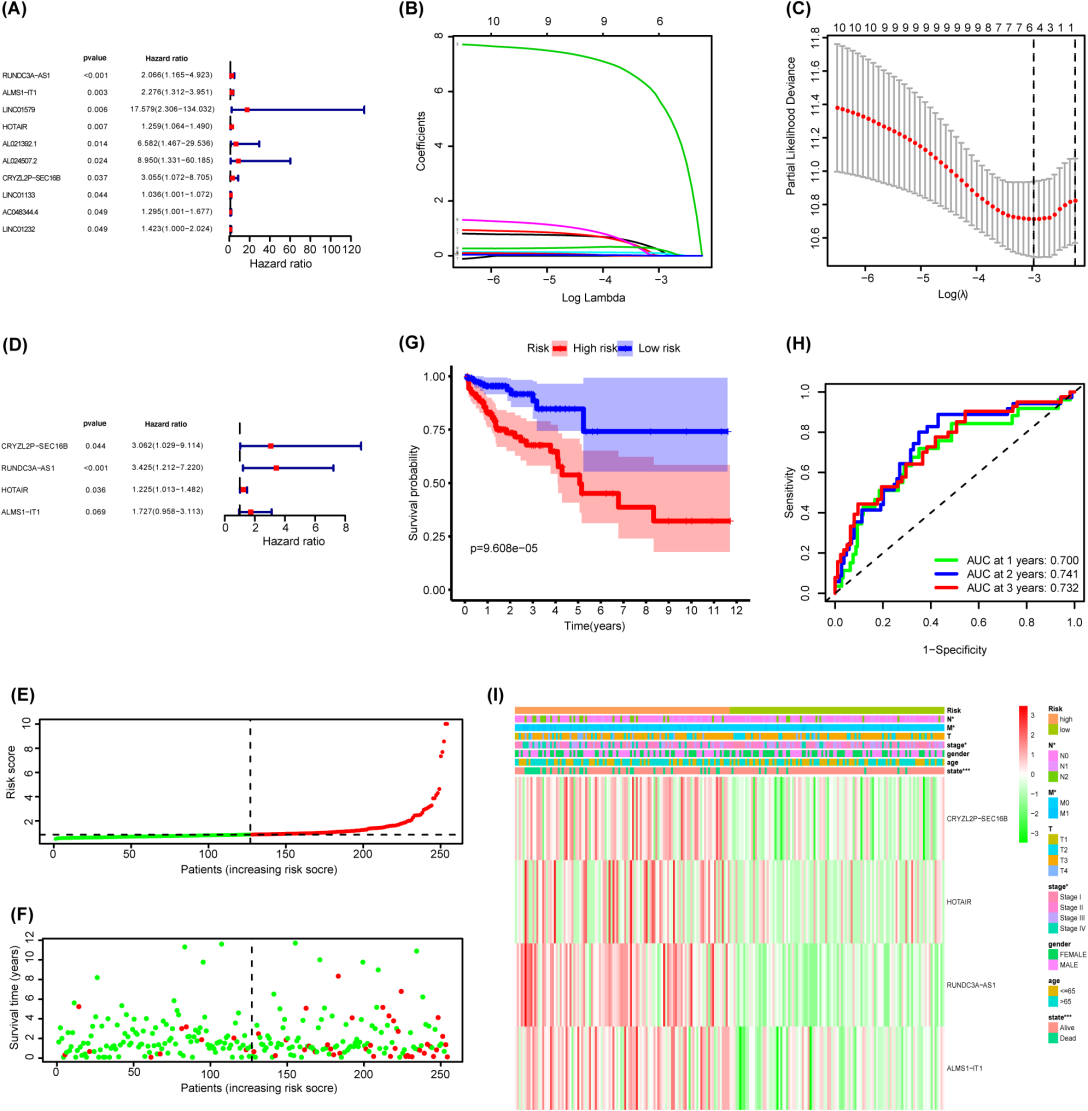
Establishment of Glycolysis-Related lncRNA Signature. **(A)** 10 glycolysis-related lncRNAs were extracted correlated with prognosis of CRC patients by univariable Cox regression analysis; **(B)** LASSO coefficient profiling; **(C)** Cross-validation for turning parameter screening in the LASSO regression model; **(D)** Multivariable Cox regression analysis was conducted to select lncRNAs independently affected OS; (**E**-**F**, **I**) The distribution of risk score, survival time and lncRNA expression of CRC patients stratified by risk score; **(G)** Kaplan-Meier survival curves for high and low-risk score groups.; **(H)** Kaplan-Meier survival analysis of CRC patients stratified by risk score; **(I)** ROC curve of predicted 1-, 2-, 3-year OS according to risk score

506 selected CRC patients were divided randomly into two equal parts, one for the training cohort, the other for internal validation cohort. 101 glycolysis-related lncRNAs were initially subjected to univariate regression analysis, with the OS of the training cohorts as the dependent outcomes. The result suggested that 10 glycolysis-related lncRNAs were significantly correlated with the OS of patients in training cohort (Fig. 3A). The LASSO regression analysis was performed to select the most valuable predictive genes with nonzero regression coefficients (Fig. 3B-C). Finally, a 4-lncRNA signature (*CRYZL2P − SEC16B, RUNDC3A − AS1, HOTAIR, and ALMS1 − IT1*) was identified using multivariate COX regression (Fig. 3D).

Then, the risk score of each patient was calculated using the formula based on the corresponding coefficients: Risk score = (expr *CRYZL2P − SEC16B* × 1.1191) + (expr *RUNDC3A − AS1* × 8.1389) - (expr *HOTAIR* × 0.2031) -(expr *ALMS1 − IT1* × 0.5462).

All patients were divided into high- or low-risk groups based on the median risk score (Fig. 3E-F). ﻿The Kaplan-Meier log-rank test confirmed that patients in the high-risk group tended to have shorter OS time in training cohort (*p* = 9.608e-05; Fig. 3G). The AUCs for 1-year, 2-year and 3-year OS were 0.7, 0.741, 0.732 ﻿respectively (Fig. 3H). The clinicopathological features were then compared between the high risk-score group and low-risk score group. We found the two groups in tumor stage (stage) (*P* < 0.05), metastasis status (M) (*P* < 0.05) and N state (*P* < 0.05) were all significantly different (Fig. 3I). We separate the patients with stage II colorectal cancer into high- or low-risk groups based on the median risk score, in training cohort (Figure S1A-B). The Kaplan-Meier log-rank test confirmed that patients with stage II colorectal cancer in different risk group have different prognosis (*P* < 0.05, Figure S1C). The AUCs for 1-year, 2-year and 3- year OS were 0.703, 0.689 and 0.637 respectively (Figure S1D).

### Construction of a nomogram

**Fig. 4:**
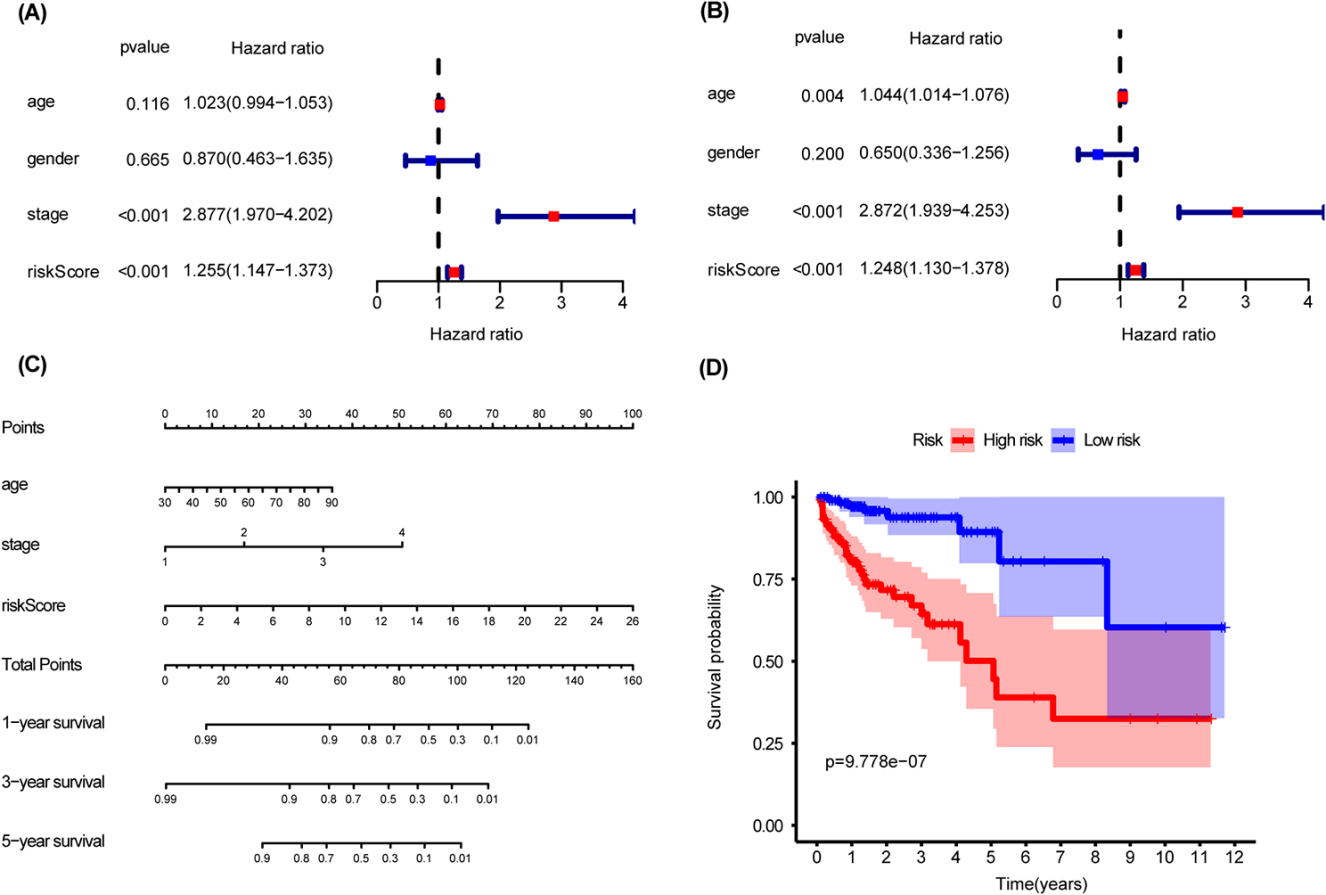
Construction of a Nomogram. (**A**-**B**) Univariable and Multivariable Cox regression analyses were performed to select independent factors influencing CRC prognosis; **(C)**The predictive nomogram; **(D)** Kaplan-Meier curves of CRC patients in train cohort stratified by the nomogram

**Fig. 5:**
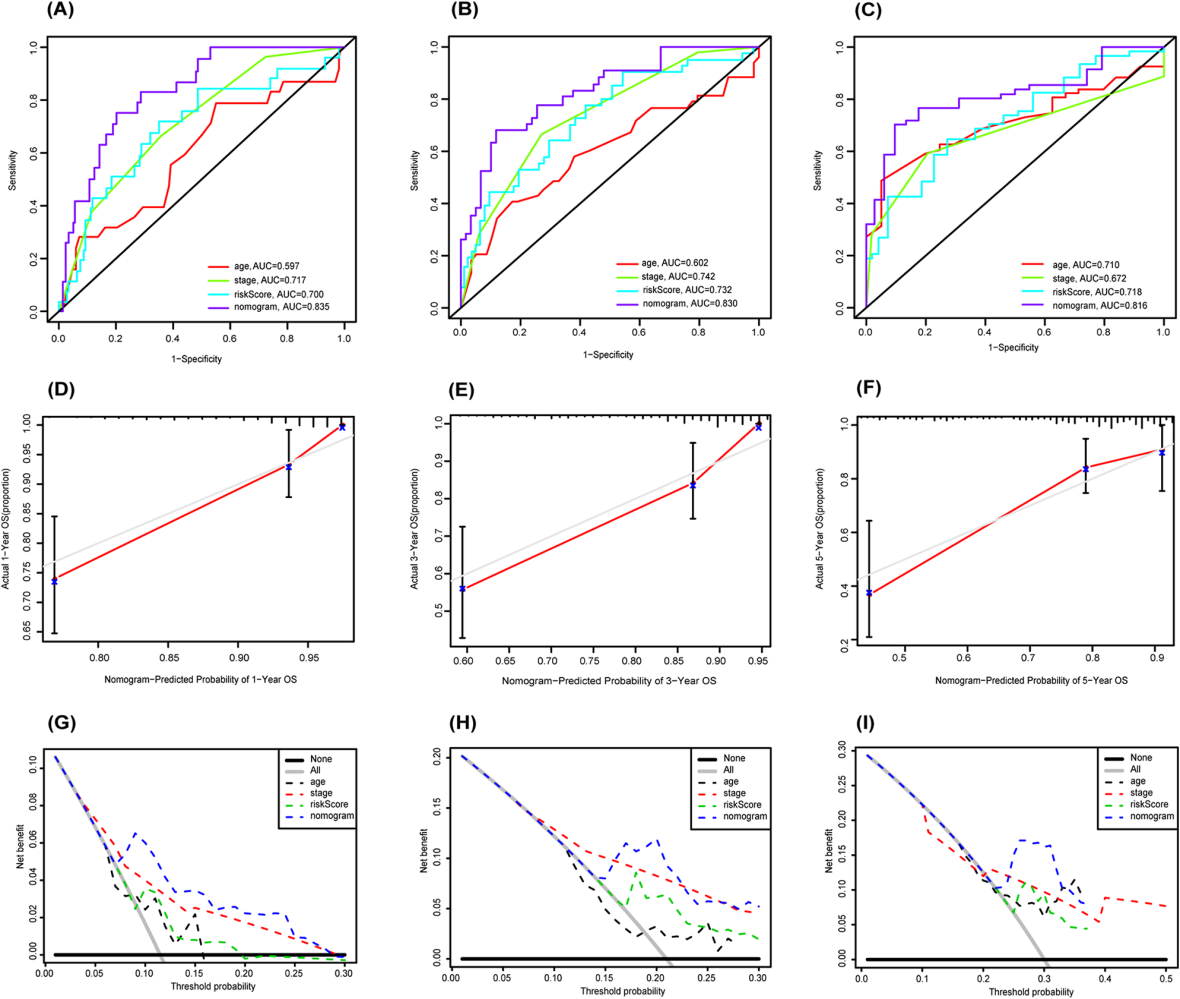
Evaluating the predictive accuracy of the nomogram. (**A**-**C**) ROC curve of predicted 1-, 3-, 5-year OS according to nomogram and other clinical characteristics. (**D**-**F**) Nomogram calibration plot of 1-, 3-, and 5-year OS in the train cohort. (**G**-**I**) Depict the 1-, 3-, and 5-year Decision Curve Analysis (DCA) curves, respectively

As tumor stage, age and gender may have an impact on the prognosis of patients with CRC. We took the factors above and the risk score as candidates consisting of nomogram. The results of univariate and multivariate Cox-regression analyses showed that, for patients with CRC, the prognostic signature (*P* < 0.001), age (*P* = 0.004) and stage (*P* < 0.001) are independent prognostic parameters (Fig. 4A-B), which we selected as the composition of the nomogram (Fig. 4C). Then, the total risk score was calculated according to each independent prognostic parameter in the nomogram. Based on the median cutoff of the total risk score we classified the patients into high- and low-risk groups. Patients in high-risk group had a poorer OS time than in the low-risk group (*P* = 9.778e − 07, Fig. 4D). We use ROC and calibration curve to evaluate the predictive accuracy of the nomogram. ROC analysis showed that the AUC values for 1-, 3-, and 5-year survival of CRC patients predicted by the total score were 0.835, 0.830, and 0.816, respectively (Fig. 5A-C), which also indicated that the nomogram had a better predictive accuracy than any of a single clinical parameter. The gray lines of the calibration curve presented the actual observation, and the red lines are the nomogram prediction of OS time (Fig. 5D-F), from which we can see a strong consistency. Similarly, the decision curves showed that the nomogram maximized clinical net benefits for patients in the prediction of OS time at 1-, 3-, 5-year (Fig. 5G-I).

### Validation of the signature and nomogram

**Fig. 6:**
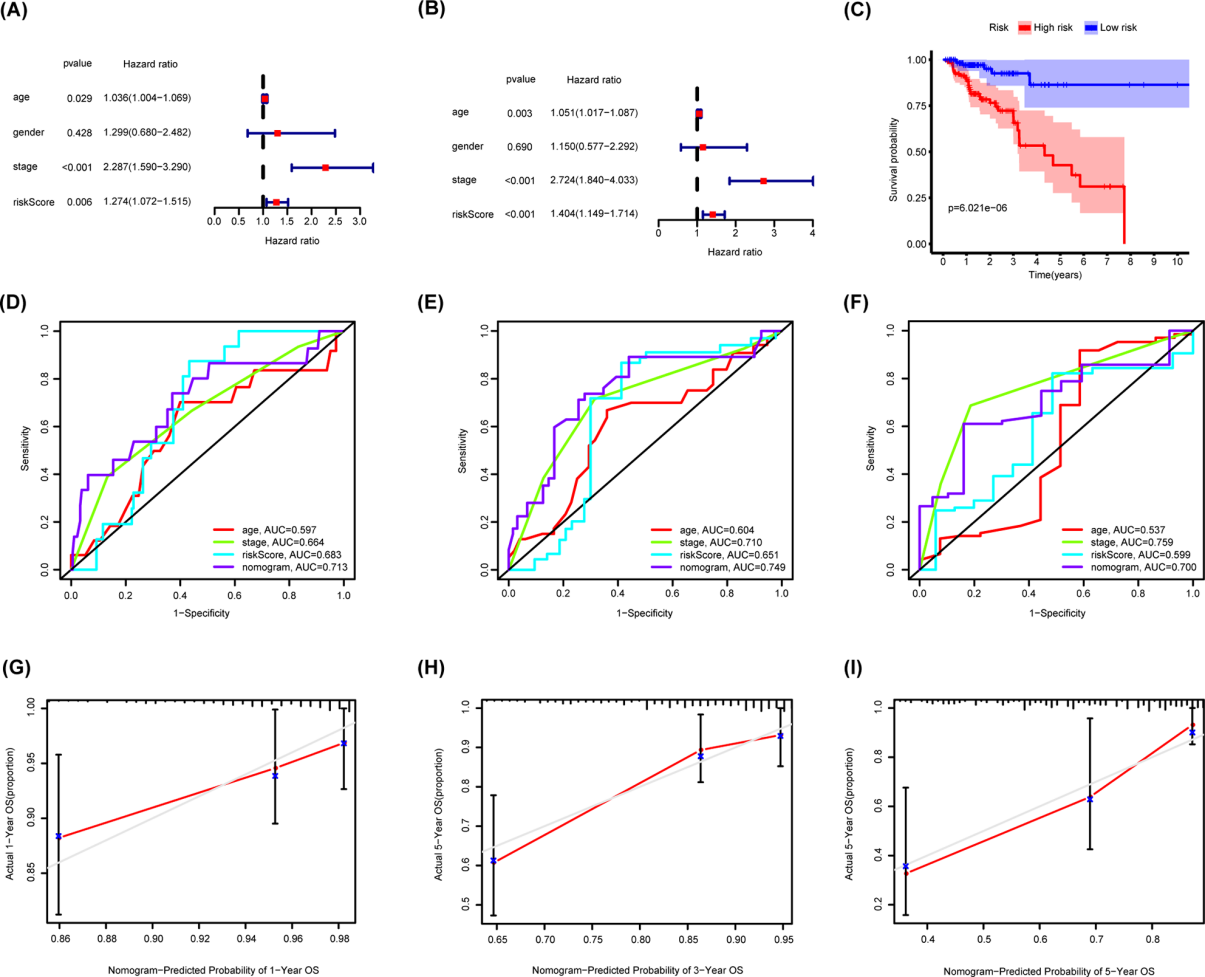
Validation of the nomogram. (**A**-**B**) Univariable and Multivariable Cox regression analyses showed that the risk signature was an independent factor for prognosis in the validation cohort; **(C)** Kaplan-Meier curves of CRC patients in the validation cohort stratified by the nomogram; (**D**-**F**) ROC curve of predicted 1-, 3-, 5-year OS according to nomogram and other clinical characteristics in the validation cohort; (**G**-**I**) Nomogram calibration plot of 1-, 3-, and 5-year OS in the validation cohort

The performance of the signature and nomogram model was validated using the validation cohort. First, the formula mentioned above was used to calculate the risk score of each patient in the internal validation cohorts. The risk score being an independent factor of the prognosis of CRC was proved again by the validation cohort (Fig. 6A-B). We separated patients in the validation cohort into high- and low- risk groups according to the same method mentioned above. The OS time of different groups showed significant difference (Fig. 6C). ROC analysis showed that the AUC values for 1-, 3-, and 5-year survival of CRC patients predicted by the total score were 0.713, 0.749, and 0.700, respectively (Fig. 6D-F) calibration curve was performed, indicating the good predictive power of the signature and nomogram (Fig. 6G-I).

Patients with stage II colorectal cancer in validation cohort were also separated into high and low-risk groups (FigureS1E-F). The OS time of different groups showed significant difference (Figure S1G). In the validation cohort, the AUCs for 1-year, 2-year and 3- year OS were 0.753, 0.702 and 0.629 respectively (Figure S1H).

### Effect of *lncRNA RUNDC3A − AS1* on CRC cells

**Fig. 7:**
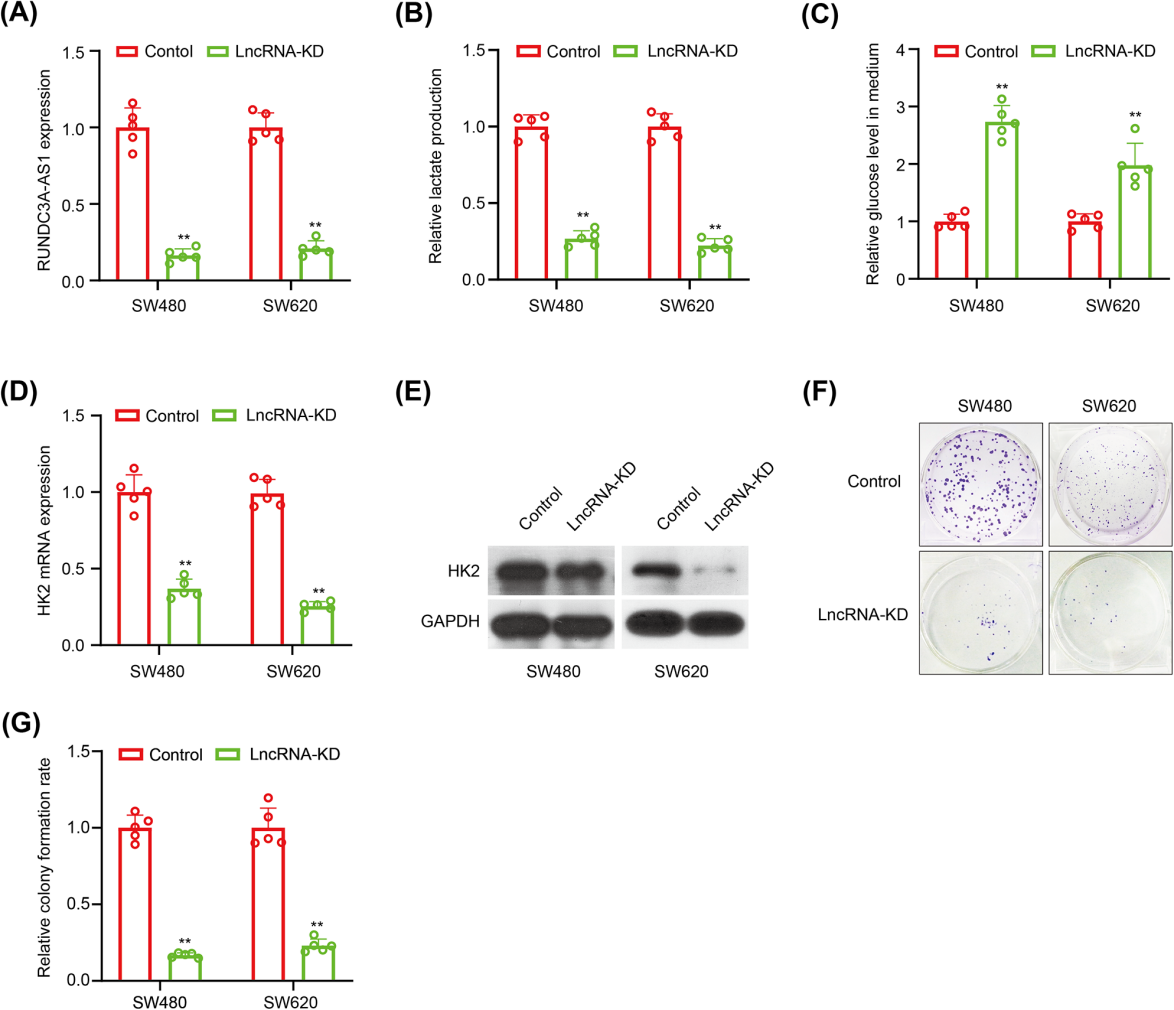
Effect of lncRNA RUNDC3A − AS1 on CRC cells. **(A)** The silence of lncRNA RUNDC3A − AS1 was achieved in SW480 and SW460 cells by transfection of si-RNA for lncRNA RUNDC3A − AS1, as confirmed by real‐time PCR; (**B**-**C**) The effects of si- RUNDC3A − AS1 inhibitor on glucose consumption and cellular lactate production levels in CRC cells; (**D**-**E**) HK2 mRNA expression and protein levels in lncRNA RUNDC3A − AS1‐silenced colorectal cancer cells examined by real‐time PCR and western-blotting analysis. (**F**-**G**) Downregulation of lncRNA *RUNDC3A − AS1* reduced the mean colony number in the colony formation assay. N_case_ = 5, N_control_ = 5

To validate the lncRNAs in the signature we constructed were glycolysis-related, we performed the glucose consumption and lactic acid production assays. In the risk score formula, lncRNA *RUNDC3A − AS1* has the maximum coefficients, indicating that the expression of lncRNA *RUNDC3A − AS1* has the highest impact among our signature on the OS of CRC patients. So, we chose to exam the effect of lncRNA *RUNDC3A − AS1* on the Warburg effect of CRC. We achieved lncRNA *RUNDC3A − AS1* knockdown to investigate its cellular function by transfecting SW480 and SW620 cells with si-*RUNDC3A − AS1*(lncRNA-KD), as confirmed by qPCR (Fig. 7A). LncRNA *RUNDC3A − AS1* silence reduced the lactate production while increased the glucose level in the CRC cells culture medium (Fig. 7B-C). Consistently, in lncRNA-KD SW480 and SW620 cells, the mRNA and protein levels of *HK2* were significantly downregulated (Fig. 7D-E), indicating a positive correlation between *HK2* and lncRNA *RUNDC3A − AS1* expression in CRC cells. In addition, we conducted colony formation assays to detect the colony formation ability of lncRNA *RUNDC3A − AS1* in colorectal cancer. Knockdown of lncRNA *RUNDC3A − AS1* significantly decreased the mean colony number in the colony formation assay (*P* < 0.001) (Fig. 7F-G). These findings indicate that *lncRNA RUNDC3A − AS1* knockdown could suppress the glycolysis and colony formation ability in CRC cells.

## Discussion

The proliferation of tumor cells fundamentally relies on an adequate supply of biological macromolecules and energy. Consequently, enhanced glycolysis emerges as a distinct hallmark of cancer cells [11]. Our research has shown that colorectal cancer (CRC) patients exhibiting significantly elevated glycolysis activity are associated with advanced tumor stages and poorer prognoses, as determined through the calculation of Gene Set Variation Analysis (GSVA) enrichment scores for each patient. Glucose serves as the primary energy substrate in the human body, metabolized through pathways including glycolysis and aerobic oxidation. During the oncogenesis and progression of CRC, notable expression changes occur in key enzymes involved in the glycolysis pathway [12]. For instance, glucose transport protein 1 (GLUT1), a key facilitator of glucose transport, is upregulated in CRC, thereby enhancing glucose absorption [13]. Conversely, the knockdown of pyruvate kinase M2 (PKM2) in cancer cells leads to a reduction in glucose uptake [14, 15]. Hexokinase II (HK2) plays a pivotal role in tumorigenesis by facilitating the Warburg effect [16]. These variations form a complex network that regulates cancer cell glucose metabolism, with cancer cells exhibiting increased glycolytic activity even in oxygen-rich environments [4]. Collectively, this evidence underscores the critical role of dysregulated glycolysis in colorectal cancer.Recent investigations have illuminated the role of long non-coding RNAs (lncRNAs) in modulating the expression of protein-coding genes and influencing cancer-related biological processes, including the Warburg effect [17–19]. Nonetheless, the mechanisms through which lncRNAs regulate the Warburg effect in colorectal cancer (CRC) remain inadequately explored. Given that one of the primary mechanisms by which lncRNAs exert their regulatory effects is through acting as endogenous microRNA (miRNA) sponges [8, 20], starting with glycolysis-related differentially expressed mRNAs to construct a competing endogenous RNA (ceRNA) network presents an efficient strategy for identifying glycolysis-associated lncRNAs. Employing this approach, we identified 101 lncRNAs potentially involved in modulating the Warburg effect in CRC, marking a pioneering effort in comprehensively screening for glycolysis-related lncRNAs.

To pinpoint lncRNAs with a strong correlation to prognosis, we conducted univariable, multivariable, and LASSO regression analyses. This process culminated in the establishment of a prognostic signature consisting of four lncRNAs (CRYZL2P − SEC16B, HOTAIR, ALMS1 − IT1, and RUNDC3A − AS1). A risk score derived from this signature was calculated, aligning with our hypothesis, emerged as an independent prognostic factor (Fig. 4). Acknowledging the significance of clinicopathological features in CRC patient outcomes, we developed a nomogram that integrates these features with our signature to enhance prognostic accuracy. The efficacy of this signature was validated through receiver operating characteristic (ROC) curves, calibration curves, and decision curves, showcasing its innovative approach in employing glycolysis-related lncRNAs. Compared to existing models [21–25], our prognostic model demonstrated superior predictive reliability in both training and validation cohorts. A notable limitation of current staging systems is their inability to account for the clinical outcome heterogeneity among patients within the same stage, especially evident in stage II CRC. Our findings reveal that the prognosis significantly diverges between high-risk and low-risk groups among stage II CRC patients based on the risk score, underscoring the utility of our signature in identifying high-risk individuals within this subgroup.Every lncRNA identified in our prognostic signature serves as an independent prognostic factor for colorectal cancer (CRC) patient outcomes. The regulatory role of lncRNA CRYZL2P − SEC16B is yet to be reported, setting a precedent for future investigations. Within our model, several lncRNAs have been previously associated with various cancers. For instance, Hu et al. highlighted that the overexpression of lncRNA HOTAIR enhances glycolysis in hepatocellular carcinoma (HCC) cells [25, 26], aligning with our predictions. ALMS1-IT1 was found by Luan et al. to potentially accelerate the malignant progression of lung adenocarcinoma by activating the cyclin-dependent kinase pathway [26, 27]. RUNDC3A − AS1 exhibited significant differential expression in papillary thyroid carcinoma, playing a crucial role in cancer histotype determination [27, 28]. Yet, its involvement in modulating the Warburg effect in cancer has not been elucidated.

In our signature, lncRNA RUNDC3A − AS1 had the highest coefficient, indicating its paramount influence on CRC prognosis. This led us to investigate RUNDC3A − AS1’s function in CRC glycolysis further. Silencing RUNDC3A − AS1 markedly reduced lactate production and glucose uptake in CRC cell lines in vitro, highlighting its potential regulatory role. Given that hexokinase, especially HK2, is pivotal in glycolysis and is overexpressed in various malignancies [15, 16, 29, 30], we examined HK2 expression post-silencing RUNDC3A − AS1. A decrease in HK2 expression in CRC cells upon downregulating RUNDC3A − AS1 underscores its probable regulatory mechanism involving HK2 and glycolysis in CRC, warranting further mechanistic studies.

In summary, our research represents the inaugural comprehensive screening of glycolysis-related lncRNAs in CRC. Utilizing these lncRNAs, we developed a four-lncRNA signature and a predictive nomogram for CRC outcomes, validated by extensive analyses for its high predictive accuracy. By downregulating lncRNA RUNDC3A − AS1, we demonstrated its significant role in modulating the Warburg effect in CRC cells, contributing novel insights into CRC glycolysis regulation.

### Electronic supplementary material

Below is the link to the electronic supplementary material.

Supplementary Material 1

## Data Availability

All data and materials are available from the corresponding author on reasonable request. Interested parties should contact Rui Mao, 218102100@csu.edu.cn for data access.

## Abbreviations

CRC: colorectal adenocarcinoma
TCGA: The Cancer Genome Atlas
GEO: Gene Expression Omnibus
ROC: receiver operating characteristic
OS: overall survival
AUC: the area under the curve
KEGG: Kyoto Encyclopedia of Genes and Genomes
qRT-PCR: Real-time quantitative reverse transcription polymerase chain reaction
