# Supplementary material for: Predictive significance of glycolysis-associated lncRNA profiles in colorectal cancer progression
Source: BMC Med Genomics. 2024 Apr 29;17:112. doi: 10.1186/s12920-024-01862-2 (PMC11057184; doi:10.1186/s12920-024-01862-2)
Supplement: Supplementary file 1 — Supplementary Material 1 [file 12920_2024_1862_MOESM1_ESM.docx]

**Supplementary Tables**

| Table S1: Top-10 up-regulated LncRNAs. | | | | | |
| --- | --- | --- | --- | --- | --- |
| Lnc-RNA | conMean | treatMean | logFC | pValue | FDR |
| AC012501.1 | 0.000547 | 0.221388 | 8.660133 | 2.06E-09 | 7.23E-09 |
| IGFL2-AS1 | 0.009511 | 0.702043 | 6.205826 | 4.54E-16 | 3.97E-15 |
| CASC19 | 0.12948 | 4.233543 | 5.031069 | 8.36E-27 | 6.72E-25 |
| CRNDE | 0.071655 | 2.231295 | 4.960679 | 1.26E-26 | 9.58E-25 |
| UCA1 | 0.781604 | 15.61061 | 4.319945 | 3.12E-16 | 2.79E-15 |
| AC092127.1 | 0.005716 | 0.112401 | 4.297514 | 0.001677 | 0.002623 |
| AC092168.2 | 0.038597 | 0.707966 | 4.197122 | 5.67E-08 | 1.64E-07 |
| H19 | 1.270988 | 21.76286 | 4.097846 | 2.46E-12 | 1.28E-11 |
| LUCAT1 | 0.024501 | 0.354119 | 3.853347 | 2.54E-15 | 2.01E-14 |
| MALAT1 | 8.033544 | 114.8 | 3.836942 | 9.09E-09 | 2.92E-08 |

Table S2: Top-10 down-regulated LncRNAs.

| Lnc-RNA | conMean | treatMean | logFC | pValue | FDR |
| --- | --- | --- | --- | --- | --- |
| CDKN2B-AS1 | 10.64837 | 0.382549 | -4.79884 | 1.02E-27 | 1.05E-25 |
| MBNL1-AS1 | 2.974191 | 0.513593 | -2.5338 | 1.24E-21 | 2.87E-20 |
| LIFR-AS1 | 0.113609 | 0.025863 | -2.13509 | 1.66E-19 | 2.49E-18 |
| HAGLR | 8.961413 | 2.205872 | -2.02238 | 2.13E-23 | 6.99E-22 |
| FENDRR | 6.221173 | 1.728232 | -1.84789 | 6.31E-22 | 1.54E-20 |
| AC010186.4 | 0.038497 | 0.011152 | -1.78748 | 6.4E-09 | 2.1E-08 |
| LINC01133 | 30.49765 | 10.47139 | -1.54224 | 2.13E-19 | 3.11E-18 |
| LINC01579 | 0.2028 | 0.085274 | -1.24989 | 0.009292 | 0.012902 |
| AP002761.4 | 3.469191 | 1.479518 | -1.22947 | 1.38E-14 | 9.84E-14 |
| AC016705.2 | 0.135499 | 0.065439 | -1.05006 | 2.97E-13 | 1.76E-12 |

**Supplementary Figures**


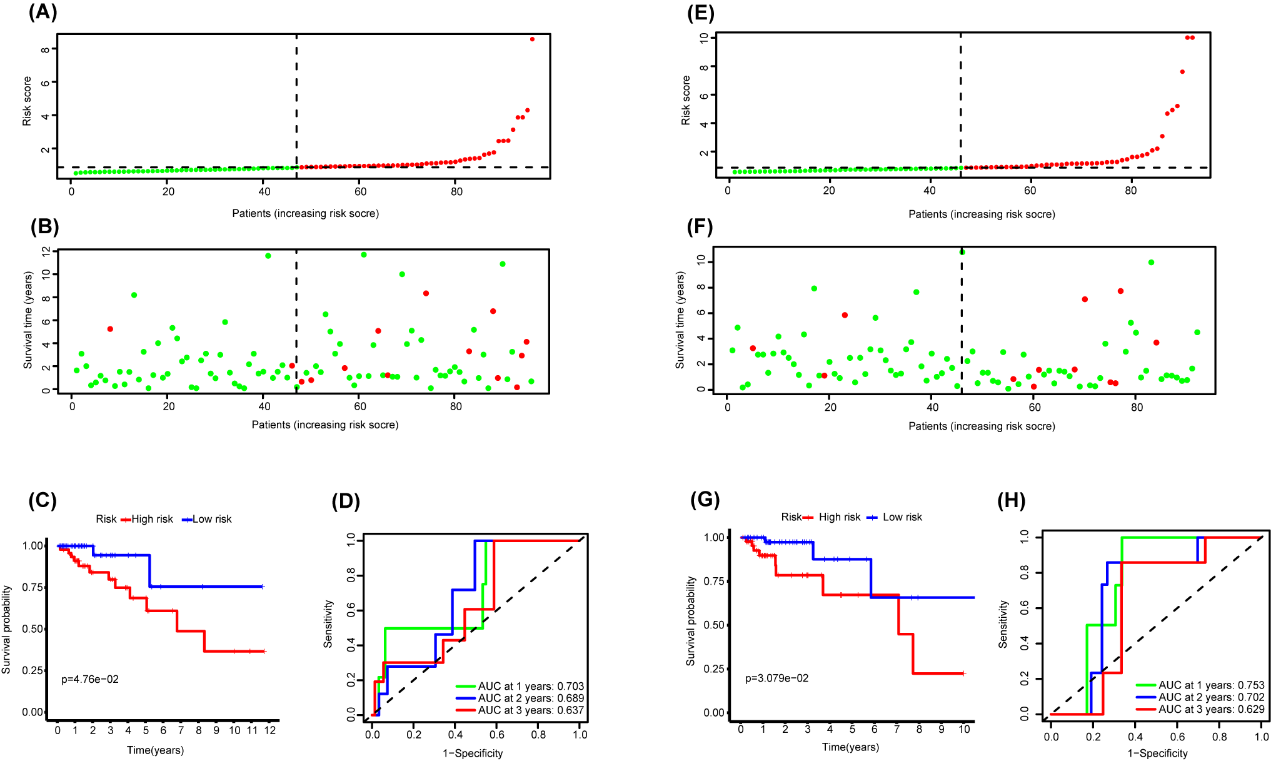


**Figure S1**: Prognosis value of glycolysis-related lncRNA signature in patients with stage II CRC. (A-B) The distribution of risk score, survival time of stage II CRC patients in training cohort stratified by risk score. (C) Kaplan-Meier curves of stage II CRC patients in training cohort. (D) ROC curve of predicted 1-, 2-, 3-year OS in stage II CRC patients according to risk score. (E-F) The distribution of risk score, survival time of stage II CRC patients in validation cohort stratified by risk score. (G) Kaplan-Meier curves of stage II CRC patients in validation cohort. (H) ROC curve of predicted 1-, 2-, 3-year OS in stage II CRC patients according to risk score.


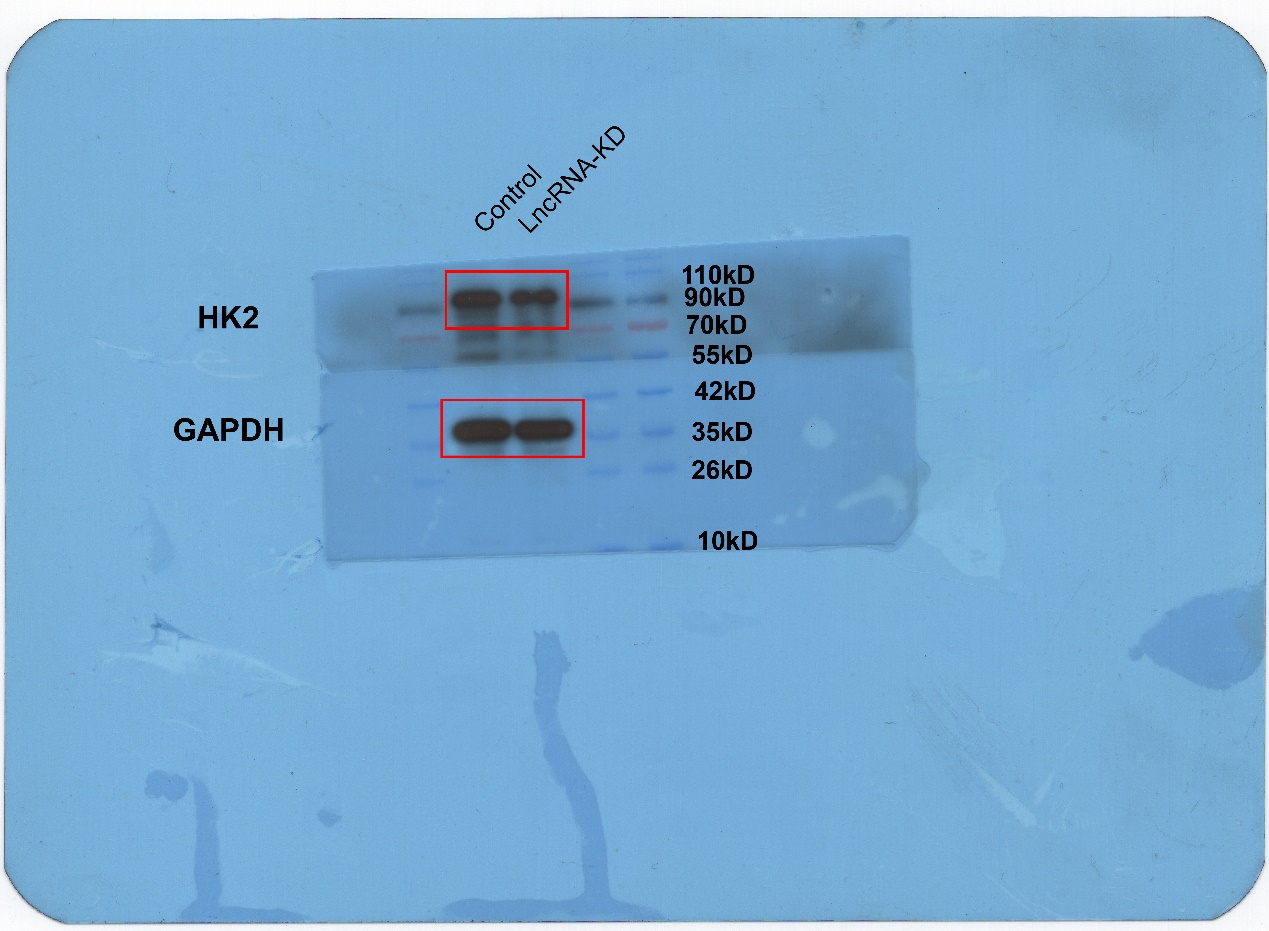

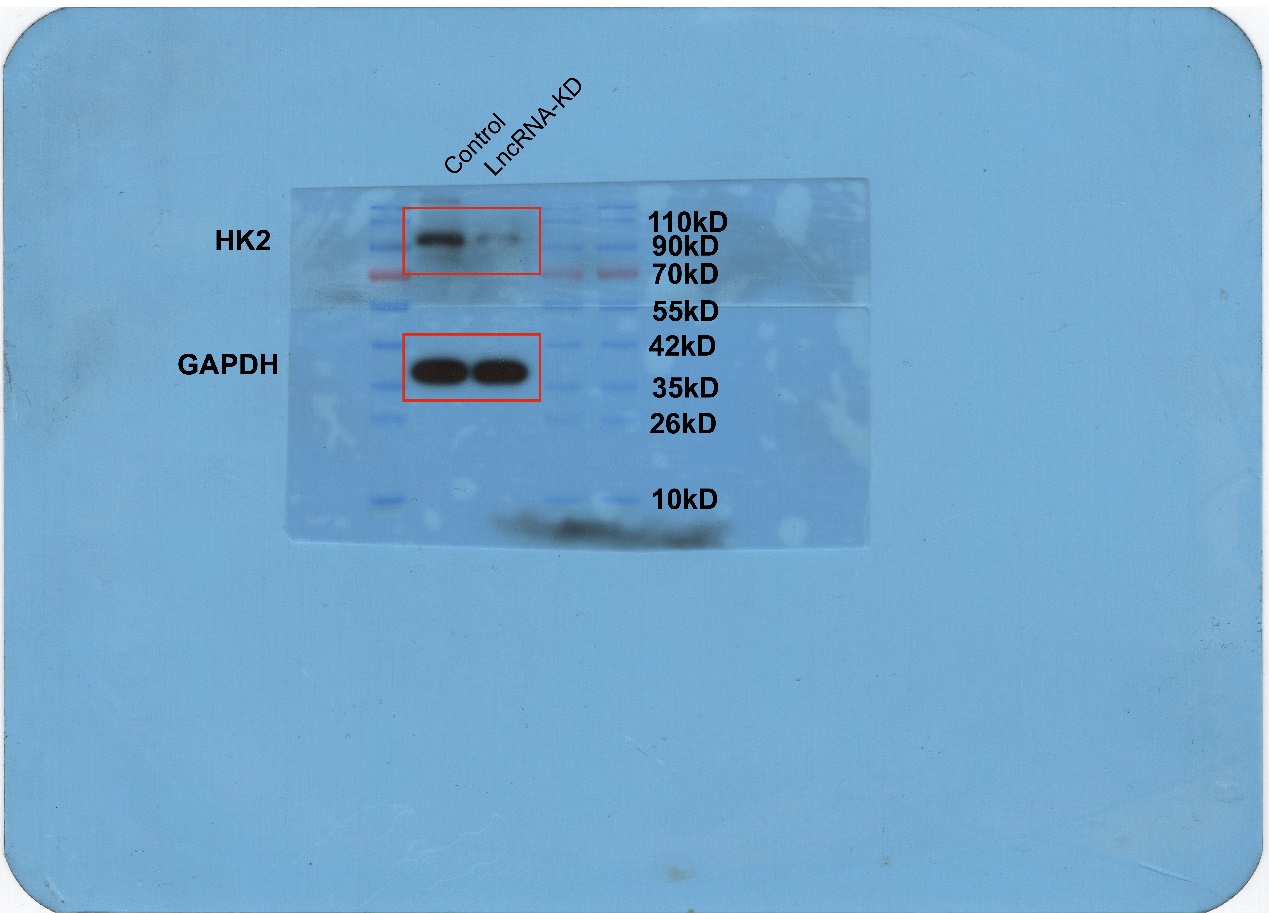


Original WB gel

R code

#cluster

library(ConsensusClusterPlus)

data <- read.table(file = "LOG2.txt", sep = "\t", header = T, stringsAsFactors = F, row.names = 1, check.names = F)

data2 <- data[apply(data, 1, function(x){sum(is.na(x)) < ncol(data)/2}),]

data2 <- as.matrix(data2)

res <- ConsensusClusterPlus(data2, maxK = 10, reps = 1000, pItem = 0.8, pFeature = 1, clusterAlg = "pam", corUse = "complete.obs", seed=123456, plot="pdf", writeTable=T)

write.table(data2, "result.txt")

#PCA

library(psych)

library(reshape2)

library(ggplot2)

library(factoextra)

library(stat)

library(vegan)

exprData <- "before.txt"

sampleFile <- "group.txt"

data <- read.table(exprData, header=T, row.names=NULL,sep="\t")

rownames_data <- make.names(data[,1],unique=T)

data <- data[,-1,drop=F]

rownames(data) <- rownames_data

data <- data[rowSums(data)>0,]

data <- data[apply(data, 1, var)!=0,]

mads <- apply(data, 1, mad)

data <- data[rev(order(mads)),]

dim(data)

data_t <- t(data)

variableL <- ncol(data_t)

if(sampleFile != "") {

sample <- read.table(sampleFile,header = T, row.names=1,sep="\t")

data_t_m <- merge(data_t, sample, by=0)

rownames(data_t_m) <- data_t_m$Row.names

data_t <- data_t_m[,-1]

}

pca <- prcomp(data_t[,1:variableL], scale=T)

print(str(pca))

library(factoextra)

fviz_eig(pca, addlabels = TRUE)

fviz_pca_ind(pca, repel=T)

fviz_pca_ind(pca, col.ind=data_t$conditions, mean.point=F, addEllipses = T, legend.title="Groups")

fviz_pca_ind(pca, col.ind=data_t$conditions, mean.point=F, addEllipses = T, legend.title="Groups", ellipse.type="confidence", ellipse.level=0.95)

fviz_pca_var(pca, select.var = list(cos2 = 0.99), repel=T, col.var = "cos2", geom.var = c("arrow", "text") )

fviz_pca_var(pca, select.var= list(cos2 = 10), repel=T, col.var = "contrib")

#Differential analysis

library(limma)

library(edgeR)

counts <- read.table(file = "conut_all.txt", sep = "\t", header = TRUE, row.names = 1, stringsAsFactors = FALSE)

dge <- DGEList(counts = counts)

dge <- calcNormFactors(dge)

logCPM <- cpm(dge, log=TRUE, prior.count=3)

group_list <- factor(c(rep("control",2), rep("siSUZ12",2)))

design <- model.matrix(~group_list)

colnames(design) <- levels(group_list)

rownames(design) <- colnames(counts)

fit <- lmFit(logCPM, design)

fit <- eBayes(fit, trend=TRUE)

output <- topTable(fit, coef=2,n=Inf)

sum(output$adj.P.Val<0.05)

#GSEA

library(clusterProfiler)

library(enrichplot)

library(ReactomePA)

library(data.table)

library("org.Hs.eg.db")

genelist_input <- fread(file="data.txt", header = T, sep='\t', data.table = F)

inputfile="gsea.txt"

gene_symbol=read.table(inputfile,sep="\t",check.names=F,header=T)

gene_name=as.vector(gene_symbol[,1])

foldChange=as.character(gene_symbol[,2])

geneID <- mget(gene_name, org.Hs.egSYMBOL2EG, ifnotfound=NA)

geneID <- as.character(geneID)

data=cbind(gene_symbol,entrezID=geneID)

head(genelist_input)

write.csv(data,"data.csv",row.names =F)

geneList = genelist_input[,2]names(geneList) = as.character(genelist_input[,1])geneList = sort(geneList, decreasing = TRUE)

Go_Reactomeresult <- gsePathway(geneList, nPerm = 1000, minGSSize = 10, maxGSSize = 1000, pvalueCutoff=0.05)

gseaplot2(Go_Reactomeresult, 1:3, pvalue_table = TRUE)

#logistics

library(plyr)

library(rms)#

library(epiDisplay)#

library(gtsummary)#

aa<-read.table("hard1.txt",header=T,sep="\t")

ddist <- datadist(aa)

options(datadist="ddist")

Uni_glm_model<-

function(x){

FML<-as.formula(paste0("status==0~",x))

glm1<-glm(FML,data=aa,family = binomial)

glm2<-summary(glm1)

OR<-round(exp(coef(glm1)),2)

SE<-glm2$coefficients[,2]

CI5<-round(exp(coef(glm1)-1.96*SE),2)

CI95<-round(exp(coef(glm1)+1.96*SE),2)

CI<-paste0(CI5,'-',CI95)

P<-round(glm2$coefficients[,4],2)

Uni_glm_model <- data.frame('Characteristics'=x,

'OR' = OR,

'CI' = CI,

'P' = P)[-1,]

return(Uni_glm_model)

}

variable.names<- colnames(aa)[c(3:26)];variable.names

Uni_glm <- lapply(variable.names, Uni_glm_model)

library(plyr)

Uni_glm<- ldply(Uni_glm,data.frame);Uni_glm

variable.names

names<- glm(status==0~age+race+marry+t+n+tnm+er+pr+her2+g+sur+rt+che,

data=aa,

family = binomial)

name<-data.frame(summary(names)$aliased)

rownames(Uni_glm)<-rownames(name)[-1]

Uni_glm <- Uni_glm[,-1]

Uni_glm$P[Uni_glm$P==0]<-"<0.001"

Uni_glm

write.csv(Uni_glm," 3.csv")

#LASSO

library(glmnet)

library(survival)

inputfile="lasso.txt"

lncRNA<-read.table(inputfile,header=T,sep="\t",row.names = 1,check.names = F,stringsAsFactors = F)

lncRNAEXP=lncRNA[,3:ncol(lncRNA)]

lncRNA=cbind(lncRNA[,1:2],lncRNAEXP)

lncRNA[,"OS"]=lncRNA[,"OS"]/365

v1<-as.matrix(lncRNA[,c(3:ncol(lncRNA))])

v2 <- as.matrix(Surv(lncRNA$OS,lncRNA$vital_status))

myfit <- glmnet(v1, v2, family = "cox")

pdf("lambda.pdf")

plot(myfit, xvar = "lambda", label = TRUE)

dev.off()

myfit2 <- cv.glmnet(v1, v2, family="cox")

pdf("min.pdf")

plot(myfit2)

abline(v=log(c(myfit2$lambda.min,myfit2$lambda.1se)),lty="dashed")

dev.off()

myfit2$lambda.1se

coe <- coef(myfit, s = myfit2$lambda.1se)

act_index <- which(coe != 0)

act_coe <- coe[act_index]

row.names(coe)[act_index]

#nomogram

library(regplot)

library("survival")

library("survminer")

tcga<-read.table("clinical.txt",header=T,sep="\t")

tcga$risk_level <- as.factor(ifelse(tcga$risk_level==1,"low","hig"))

res.cox <- coxph(Surv(OS, vital_status) ~ age + sex + stage + risk_score, data = tcga)

res.cox

summary(res.cox)

nom1<-regplot(res.cox, clickable=TRUE,

points=TRUE, rank="sd",failtime = c(1095,1825,2555),prfail = T)

nom2<-regplot(res.cox,observation=tcga[5,], clickable=TRUE,

points=TRUE, rank="sd",failtime = c(1095,1825,2555),droplines=T,prfail = T,

other=(list(bvcol="red",sq="green",obscol="blue")))

#nomogram

library(regplot)

library(rms)

library(rmda)

non_tumor<-read.table("ROC.txt",header=T,sep="\t")

non_tumor$sex <- as.factor(ifelse(non_tumor$sex==1,"male","female"))

ddist <- datadist(non_tumor)

options(datadist="ddist")

mylog <-lrm(status ~ HMGCR + ACSS2 + CUX2 + PNPLA3, family=binomial(link = "logit"), data =non_tumor)

mylog

summary(mylog)

coefficients(mylog)

exp(coefficients(mylog))

exp(confint(mylog))

nom1<-regplot(mylog, clickable=TRUE,

points=TRUE, rank="sd",prfail = T)

#指定标记的样本行

nom2<-regplot(mylog,observation=non_tumor[53,], clickable=TRUE,

points=TRUE, rank="sd",droplines=T,prfail = T,

other=(list(bvcol="red",sq="green",obscol="blue")))

mylog<-lrm(status~HMGCR + PLIN2 + CUX2 + PNPLA3 + FAR2 + UBIAD1 + ACOX2 + SYNE4 + CLMP + KRT4 + ACSS2 + PCCB + MSMO1 + RMDN3 + IDH1 ,data=non_tumor,x=T,y=T)

mylog<-lrm(status~HMGCR + PLIN2 + CUX2 + PNPLA3 ,data=non_tumor,x=T,y=T)

mynom<- nomogram(mylog, fun=plogis,fun.at=c(0.0001,0.1,0.2,0.3,0.4,0.5,0.6,0.7,0.8,0.9,0.9999),lp=F, funlabel="risk of PSD")

pdf("Nom_2.pdf",10,8)

plot(mynom)

dev.off()

mylog<-lrm(status~history_of_hyperlipidemia + NIHSS + Time_from_onset_to_hospitalization + cultural_level,data=non_tumor,x=T,y=T)

mylog

library(Hmisc)

Cindex <- rcorrcens(non_tumor$status~predict(mylog))

Cindex

mylog<-lrm(status~TMEM97 + HILPDA + CUX2 + DHCR7,data=non_tumor,x=T,y=T)

set.seed(300)

myc<-validate(mylog,method="b",B = 1000,pr=T,dxy=T)

c_index<-(myc[1,5]+1)/2

c_index

mylog<-lrm(status~TMEM97 + HILPDA + CUX2 + DHCR7,data=non_tumor,x=T,y=T)

mycal<-calibrate(mylog,method="boot",B=1000)

pdf("Calibration_2.pdf")

plot(mycal,xlab="Nomogram-predicted probability of PSD",ylab="Actual diagnosed PSD (proportion)",sub=T)

dev.off()

nom1<-regplot(mylog, clickable=TRUE,

points=TRUE, rank="sd",prfail = T)

nom2<-regplot(mylog,observation=non_tumor[5,], clickable=TRUE,

points=TRUE, rank="sd",droplines=T,prfail = T,

other=(list(bvcol="red",sq="green",obscol="blue")))

modul<- decision_curve(status~TMEM97 + HILPDA + CUX2 + DHCR7,data= non_tumor,

family = binomial(link ='logit'),

thresholds= seq(0,1, by = 0.01),

confidence.intervals = 0.95)

pdf("DCA2.pdf")

plot_decision_curve(modul,

curve.names="cALL nomogram",xlab="Threshold probability",

cost.benefit.axis =FALSE,col= "gray30",

confidence.intervals=FALSE,

standardize = FALSE)

dev.off()

modul1<- decision_curve(status~

,data= non_tumor,

family = binomial(link ='logit'),

thresholds= seq(0,1, by = 0.01),

confidence.intervals = 0.95)

modul2<- decision_curve(status~

,data= non_tumor,

family = binomial(link ='logit'),

thresholds= seq(0,1, by = 0.01),

confidence.intervals = 0.95)

modul3<- decision_curve(status~

,data= non_tumor,

family = binomial(link ='logit'),

thresholds= seq(0,1, by = 0.01),

confidence.intervals = 0.95)

modul4<- decision_curve(status~

,data= non_tumor,

family = binomial(link ='logit'),

thresholds= seq(0,1, by = 0.01),

confidence.intervals = 0.95)

modul5<- decision_curve(status~

,data= non_tumor,

family = binomial(link ='logit'),

thresholds= seq(0,1, by = 0.01),

confidence.intervals = 0.95)

pdf("DCA1.pdf")

plot_decision_curve(list(modul1,modul2,modul3,modul4,modul5),

curve.names= c("complete nomogram","HRH1","HRH2","HTR3A","TRPV2"), xlab="Threshold probability",

cost.benefit.axis =FALSE,col=c( "Orange","HotPink","Turquoise","red","green"),

confidence.intervals=FALSE,

standardize = FALSE)

dev.off()

#risk score

library(survival)

library(rms)

library(foreign)

tcga<-read.table("clinical.txt",header=T,sep="\t")

ddist <- datadist(tcga)

options(datadist='ddist')

cox2 <- coxph(Surv(OS,vital_status) ~ age + sex + stage + risk_score,data=tcga)

risk_score<-predict(cox2,type="risk",newdata=tcga)

risk_level<-as.vector(ifelse(risk_score>median(risk_score),"High","Low"))

write.table(cbind(id=rownames(cbind(tcga[,1:2],risk_score,risk_level)),cbind(tcga[,1:2],risk_score,risk_level)),"risk_score.txt",sep="\t",quote=F,row.names=F)

#Forest plot

setwd("")

rt <- read.table("mulcox.txt",header=T,sep="\t",row.names=1,check.names=F)

gene <- rownames(rt)

hr <- sprintf("%.3f",rt$"HR")

hrLow <- sprintf("%.3f",rt$"HR.95L")

hrHigh <- sprintf("%.3f",rt$"HR.95H")

Hazard.ratio <- paste0(hr,"(",hrLow,"-",hrHigh,")")

pVal <- ifelse(rt$pvalue<0.001, "<0.001", sprintf("%.3f", rt$pvalue))

pdf(file="forest-mulcox.pdf", width =6,height =5)

n <- nrow(rt)

nRow <- n+1

ylim <- c(1,nRow)

layout(matrix(c(1,2),nc=2),width=c(3,2))

xlim = c(0,3)

par(mar=c(4,2.5,2,1))

plot(1,xlim=xlim,ylim=ylim,type="n",axes=F,xlab="",ylab="")

text.cex=0.8

text(0,n:1,gene,adj=0,cex=text.cex)

text(1.5-0.5*0.2,n:1,pVal,adj=1,cex=text.cex);text(1.5-0.5*0.2,n+1,'pvalue',cex=text.cex,font=2,adj=1)

text(3,n:1,Hazard.ratio,adj=1,cex=text.cex);text(3,n+1,'Hazard ratio',cex=text.cex,font=2,adj=1,)

par(mar=c(4,1,2,1),mgp=c(2,0.5,0))

xlim = c(0,max(as.numeric(hrLow),as.numeric(hrHigh)))

plot(1,xlim=xlim,ylim=ylim,type="n",axes=F,ylab="",xaxs="i",xlab="Hazard ratio")

arrows(as.numeric(hrLow),n:1,as.numeric(hrHigh),n:1,angle=90,code=3,length=0.05,col="darkblue",lwd=2.5)

abline(v=1,col="black",lty=2,lwd=2)

boxcolor = ifelse(as.numeric(hr) > 1, 'red', 'green')

points(as.numeric(hr), n:1, pch = 15, col = boxcolor, cex=1.3)

axis(1)

dev.off()
